# Impact of data preprocessing on cell-type clustering based on single-cell RNA-seq data

**DOI:** 10.1186/s12859-020-03797-8

**Published:** 2020-10-07

**Authors:** Chunxiang Wang, Xin Gao, Juntao Liu

**Affiliations:** 1 School of Mathematics and Statistics, Shandong University (Weihai), Weihai, 264209 China; 2 Computer, Electrical and Mathematical Sciences and Engineering Division, Computational Bioscience Research Center (CBRC), King Abdullah University of Science and Technology (KAUST), Thuwal, 23955 Saudi Arabia

**Keywords:** Preprocessing method, Single-cell RNA-seq data, Gene expression data, Single-cell clustering, SC3

## Abstract

**Background:**

Advances in single-cell RNA-seq technology have led to great opportunities for the quantitative characterization of cell types, and many clustering algorithms have been developed based on single-cell gene expression. However, we found that different data preprocessing methods show quite different effects on clustering algorithms. Moreover, there is no specific preprocessing method that is applicable to all clustering algorithms, and even for the same clustering algorithm, the best preprocessing method depends on the input data.

**Results:**

We designed a graph-based algorithm, SC3-e, specifically for discriminating the best data preprocessing method for SC3, which is currently the most widely used clustering algorithm for single cell clustering. When tested on eight frequently used single-cell RNA-seq data sets, SC3-e always accurately selects the best data preprocessing method for SC3 and therefore greatly enhances the clustering performance of SC3.

**Conclusion:**

The SC3-e algorithm is practically powerful for discriminating the best data preprocessing method, and therefore largely enhances the performance of cell-type clustering of SC3. It is expected to play a crucial role in the related studies of single-cell clustering, such as the studies of human complex diseases and discoveries of new cell types.

## Background

Single-cell RNA sequencing (scRNA-seq) has revolutionized traditional transcriptomic studies by extracting the transcriptome information at the resolution of a single cell; therefore, this approach is able to detect heterogeneous information that cannot be obtained by sequencing mixed cells and to reveal the genetic structure and gene expression status of a single cell [1–7]. Moreover, it helps to identify new cell types [8, 9], provides new research ideas and opens up new directions for in-depth research on the occurrence, development mechanisms, diagnosis and treatment of complex diseases [10]. However, scRNA-seq generally results in a large amount of noise, and the capture efficiency is also much lower than that of traditional bulk RNA-seq, generating a very large number of dropouts, which gives rise to new challenges in single-cell data analysis and calculation [11]. Accordingly, use of unsupervised clustering algorithms based on such noisy single-cell gene expression data has become the main computational strategy for identifying cell types, which is usually the first step for the subsequent analysis of scRNA-seq data (e.g., the reconstruction of cell developmental trajectories) [12, 13].

A number of clustering methods have been developed by using scRNA-seq data; e.g., Xu and Su designed a new method by using a shared nearest neighbor approach followed by a quasi-clique-based clustering algorithm (SNN-cliq) to cluster single-cell transcriptomes [14]. In addition, the approach that uses a shared nearest neighbor approach followed by walktrap was applied for cell type clustering [15]. The DynamicTreecut method was designed by using a voting strategy based on approval votes from known markers [16]. The SC3 algorithm [17] performs cell-type clustering using a strategy combining multiple clustering solutions to generate a consensus result. Clustering methods such as tSNE [18] followed by k-means (tSNE + kmeans which was also tested in the study [17]) and pcaReduce [19] perform dimensionality reduction before clustering to extract principal components and reduce computational complexity. Among these methods, SC3 is the most widely used clustering method with high accuracy and adaptability, mainly because of its consensus strategy. Although great efforts have been made in the development of these clustering algorithms to effectively cluster cell types, the noise caused by artifacts induced by laboratory protocols during single-cell sequencing and the lack of the universality of the clustering algorithms themselves mean that the clustering accuracy is far from sufficient for many practical applications, and there remains a large amount of room for the improvement of clustering models.

Generally, most single-cell clustering methods use gene expression data as their input, estimated from the scRNA-seq data of individual cells. A critical step in those clustering methods is to perform data preprocessing before cell-type clustering to eliminate the effects of confounding factors and reduce the effects of noise in the sample. Gene expression data record the expression value of each gene in each cell generally by transcripts per million mapped reads (TPM), counts per million mapped reads (CPM), reads per kilobase of transcript per million mapped reads (RPKM), fragments per kilobase of transcript per million mapped reads (FPKM), read counts mapped to a gene (READS), quantile normalization (QN) or others. For a given gene expression data set, the commonly used data preprocessing methods for single-cell clustering include log transformation, z-score transformation and a newly developed approach sctransform [20] in the statistical sense [21]. Then, a challenging problem for a specific single-cell gene expression data set is whether preprocessing should be performed for the given data before clustering. If the answer is yes, which kind of preprocessing method should we choose?

In this study, we analyzed the effects of data preprocessing on clustering results in detail by applying several widely employed clustering methods, such as SC3, dynamicTreecut, pcaReduce, tSNE + *k*-means, and SNN-clip, to eight commonly used single-cell gene expression data sets. The results showed that different data preprocessing methods have quite different effects on different clustering algorithms for different types of gene expression data. Additionally, some clustering methods showed the best clustering results for certain data sets without any preprocessing. Therefore, we conclude that there is no specific preprocessing approach that is applicable to all clustering methods for any gene expression data set. Based on this conclusion, we designed the graph-based SC3-e algorithm specifically for discriminating the best data preprocessing method for SC3 algorithm. When our algorithm was tested on the eight frequently used single-cell gene expression data sets, SC3-e always accurately selects the best preprocessing method for SC3 and therefore greatly enhances the performance of SC3.

## Results

### Impact of different preprocessing methods on cell-type clustering

In this study, five commonly used clustering methods (dynamicTreecut, tSNE + *k*-means, SNN-clip, pcaReduce, and SC3) were applied to evaluate clustering performance under four of the most commonly used data preprocessing methods (log transformation, z-score transformation, no transformation, and sctransform) with eight frequently used data sets (see “Methods” section for the details of the eight data sets, Darmanis, Lake, Yan, Romanov, Baron, Biase, Deng, and Leng). The clustering accuracy was evaluated by the commonly used criterion of the adjusted Rand index (ARI), as defined in the “Methods” section.

After running the five clustering algorithms under the four preprocessing methods on all eight data sets, the results showed that different preprocessing methods have quite different effects on the five clustering algorithms, and none of the four preprocessing methods is applicable to all clustering methods for any gene expression data set. As shown in Table 1, dynamicTreecut performed the best under log transformation for six of the data sets, but performed the best for three data sets under sctransform. PcaReduce showed its best performance for four data sets under sctransform, tSNE + *k*-means also showed its best performance for five data sets under log transformation, while pcaReduce performed best for three data sets under log transformation, and tSNE + *k*-means performed best for three data sets under sctransform. SNN-clip showed its best performance under z-score transformation for three data sets, while it also worked best under sctransform for three data sets. SC3 showed its best performance for three data sets under z-score transformation, while it worked best under no transformation for two data sets, and it worked best under sctransform for two data sets. Based on the above results, it is clear that different clustering methods are quite differently affected by different preprocessing methods. Even for the same clustering algorithm, the best preprocessing method still depends on the input data. Log transformation and sctransform seem to be applicable to most clustering methods for a large number of data sets, and z-score transformation also performs the best for SNN-clip and SC3 for multiple data sets.

**Table 1:** ARI values of the five clustering algorithms under the four preprocessing methods

	Darmanis	Lake	Yan	Baron	Biase	Leng	Romanov	Deng
dynamicTreecut								
sctransform	0.368	0.195	**0.667**	0.376	*	0.069	**0.562**	**0.858**
log	**0.37**	0.206	**0.667**	**0.629**	**1**	**0.101**	0.51	**0.858**
no	0.28	**0.21**	**0.667**	0.16	0.71	0.052	0.191	0.582
z-score	0.015	0.003	0.296	0	0.241	0	0	0.018
pcaReduce								
sctransform	0.457	**0.292**	**0.818**	**0.425**	*	0.265	**0.379**	0.361
log	**0.46**	0.29	0.779	0.415	**0.388**	**0.258**	0.364	0.409
no	0.071	0.156	0.41	0.278	0.004	0.154	0.166	0.422
z-score	0.442	0.276	0.671	0.238	0.325	0.056	0.221	**0.518**
tSNE + k-means								
sctransform	0.467	**0.304**	0.679	**0.462**	*	0.059	0.429	**0.481**
log	**0.479**	**0.304**	**0.684**	0.445	**0.772**	0.055	**0.44**	0.449
no	0.43	0.276	0.618	0.311	0.76	**0.126**	0.29	0.435
z-score	0.034	0.01	0.351	0.03	0.002	0.015	0.098	0.156
SNN-clip								
sctransform	0.572	**0.52**	0.673	**0.515**	*	0.26	**0.422**	0.594
log	0.609	0.501	0.673	0.477	**0.581**	0.241	0.387	0.596
no	0.077	0.249	0.722	0.326	0.476	0.252	0.279	0.576
z-score	**0.643**	0.498	**0.744**	0.395	0.216	**0.277**	0.228	0.483
SC3								
sctransform	**0.795**	**0.556**	0.658	0.537	*	0.21	0.519	0.571
log	0.785	0.554	0.674	0.56	0.87	0.22	0.511	0.575
no	0.492	0.415	0.595	**0.757**	0.783	0.221	**0.575**	**0.841**
z-score	0.656	0.494	**0.895**	0.489	**0.956**	**0.594**	0.336	0.686

Each bold number in the table represents the maximum ARI of a clustering algorithm.

Moreover, for a given data set, there can be large differences between clustering accuracies under different preprocessing methods. For example, the clustering accuracy of SC3 under z-score transformation for the Leng data set was 0.594, while it was only 0.21, 0.22 and 0.221 under log, no transformation and sctransform, respectively. Similarly, the clustering accuracy of dynamicTreecut under log transformation, no transformation, and sctransform for the Yan data set was 0.667, while the accuracy was only 0.296 under z-score transformation. The default preprocessing method of SC3 is log transformation, which is not the optimal method for any of the tested datasets. By selecting the best preprocessing method for different data sets, the ARI of the SC3 clustering result will be increased by up to 37%. Therefore, the choice of the best preprocessing method would greatly improve the performance of the clustering methods.

### Performance evaluation of the SC3-e algorithm

Based on the above conclusions, we designed a graph-based algorithm, SC3-e, specifically for discriminating the optimal data preprocessing method for the SC3 algorithm. It first builds two new graph models for each preprocessing method by using the clustering result and the corresponding consensus matrix generated by SC3, based on which a so-called C-score value can be calculated for each preprocessing method. And then the optimal preprocessing method for the SC3 clustering algorithm can be effectively and stably determined by the C-score values.

#### Validity of SC3-e to discriminate the best preprocessing method

**Fig. 1:**
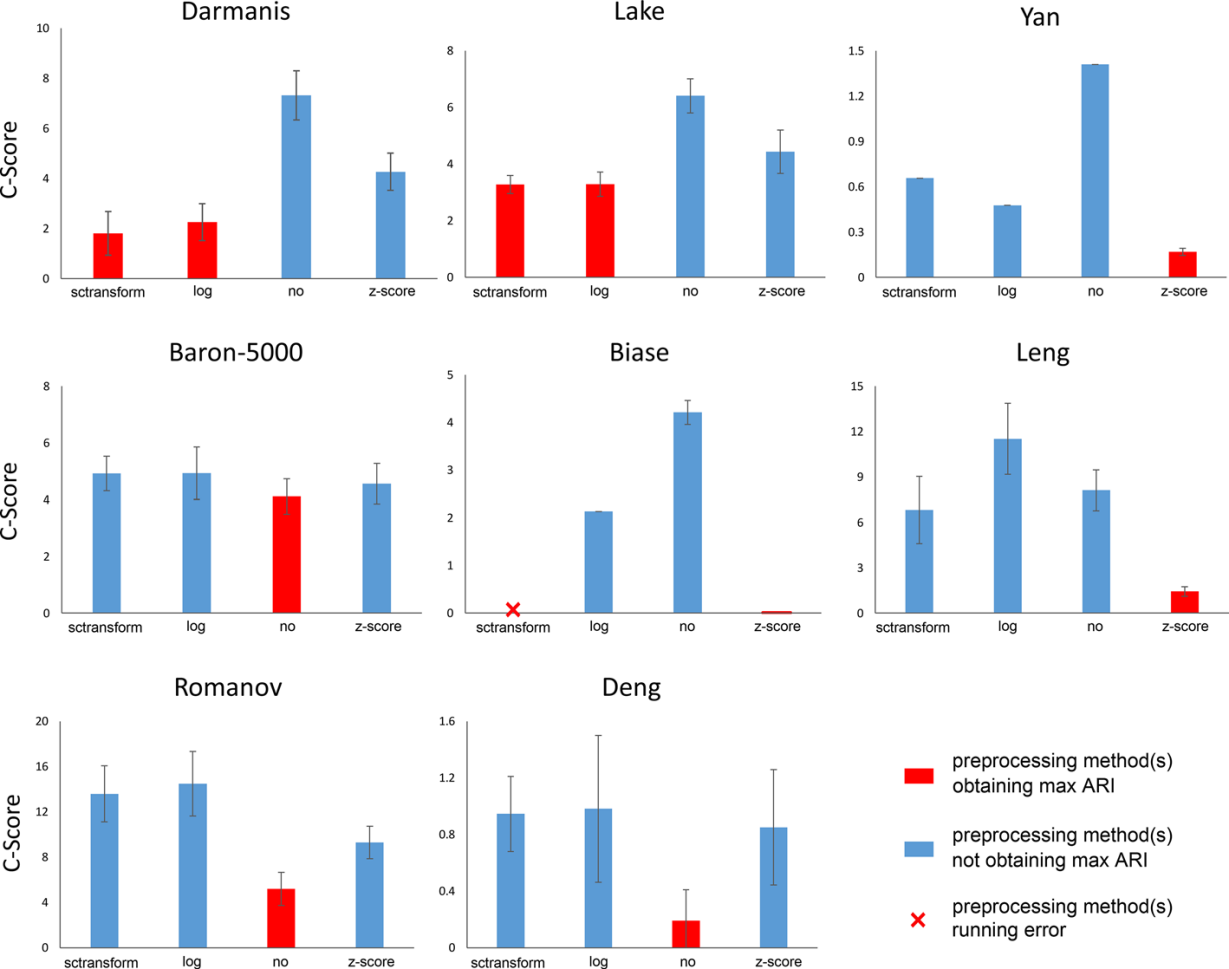
Error bars of C-scores of SC3-e obtained via 50 runs for each preprocessing method for the eight data sets. Each bar represents the values of the corresponding 50 C-scores

After running SC3-e on each data set, we obtained four C-scores (see details in the “Selection of the best preprocessing method for SC3” section) corresponding to the four preprocessing methods of log transformation, z-score transformation, no transformation, and sctransform, and the smaller the C-score, the better the corresponding preprocessing method. To test the validity of SC3-e, we ran SC3-e 50 times on each of the eight data sets, and the results showed that SC3-e effectively discriminated the best preprocessing method from the other three preprocessing methods in most cases (see Fig. 1). As a result of the instability of SC3, the 50 calculated C-scores may be different, and the best preprocessing method will sometimes generate a larger C-score. However, the trend of the best preprocessing method generating the smallest C-score was always maintained.

In addition, we calculated the average value of the 50 C-scores (see Fig. 1), which demonstrated that the best preprocessing method always produced the smallest average C-score value. Therefore, the C-score value is effective in discriminating the best preprocessing methods for the SC3 clustering algorithm in practical applications. In addition, we did a correlation analysis between the two values C-score and ARI on all the eight data sets by using Pearson correlation coefficient, and results showed that the Pearson correlation coefficients were − 0.8982, − 0.8272, − 0.8206, − 0.3065, − 0.9927, − 0.7864, − 0.0214, and − 0.5724 respectively on the eight data sets. According to the results, the two values C-score and ARI showed high correlation on most data sets. For the reason why C-score is significantly less predictive of clustering quality on some data sets, it may be that these data sets (e.g. the Baron-5000 and Romanov data sets) contain higher number of cells, which makes the constructed consensus matrix and the final clustering result by SC3 more instable.

#### Evaluation of the stability of SC3-e algorithm

To evaluate the stability of the SC3-e algorithm, we first ran SC3-e by using different values of the *N* parameter (repeat number for calculating C-scores as described in the “Methods” section) for all eight data sets, and for each *N*, SC3-e was run 100 times. In each SC3-e run, if the best preprocessing method is correctly discriminated, that run is referred to as a *good run*. Then, the *accuracy* was defined as the fraction of good runs out of all 100 runs.

**Fig. 2:**
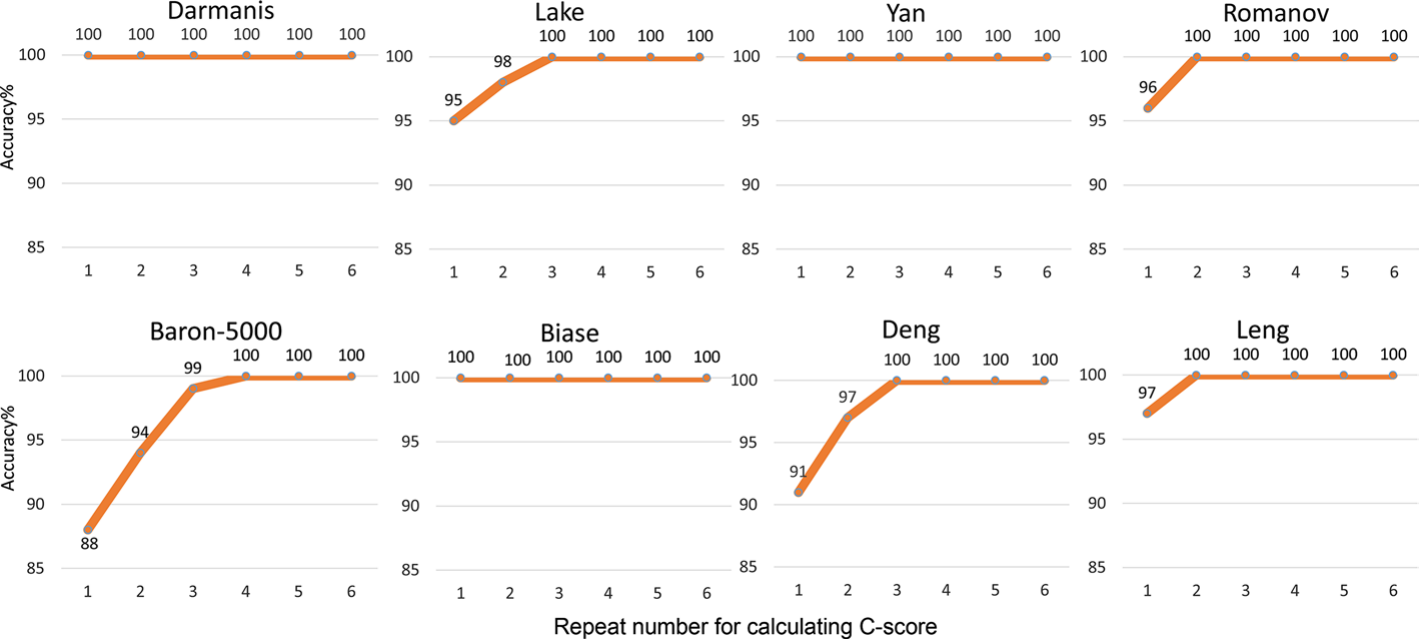
Accuracy of SC3-e for the eight data sets. The abscissa axis in each plot represents the repeat number, *N*

After running SC3-e on all the eight data sets, the results showed that the parameter *N* set to 3 would generate 100% accuracy for seven data sets, with the exception of the Baron-5000 data set, for which the accuracy was 99% when *N* was set to 3 and 100% when *N* was set to 4. Therefore, SC3-e generally only needs to be repeated 3 times to select the best preprocessing method for SC3 (see Fig. 2). In fact, for most of the tested data sets, setting the value of *N* to 1 would produce very high accuracy (more than 90% for seven data sets); therefore, the default value of *N* is set to 1 in the design of SC3-e algorithm. Based on the above evaluations, we concluded that SC3-e is not only effective but also quite stable.

#### Performance comparisons between SC3-e and the other clustering algorithms by using the best preprocessing methods

**Fig. 3:**
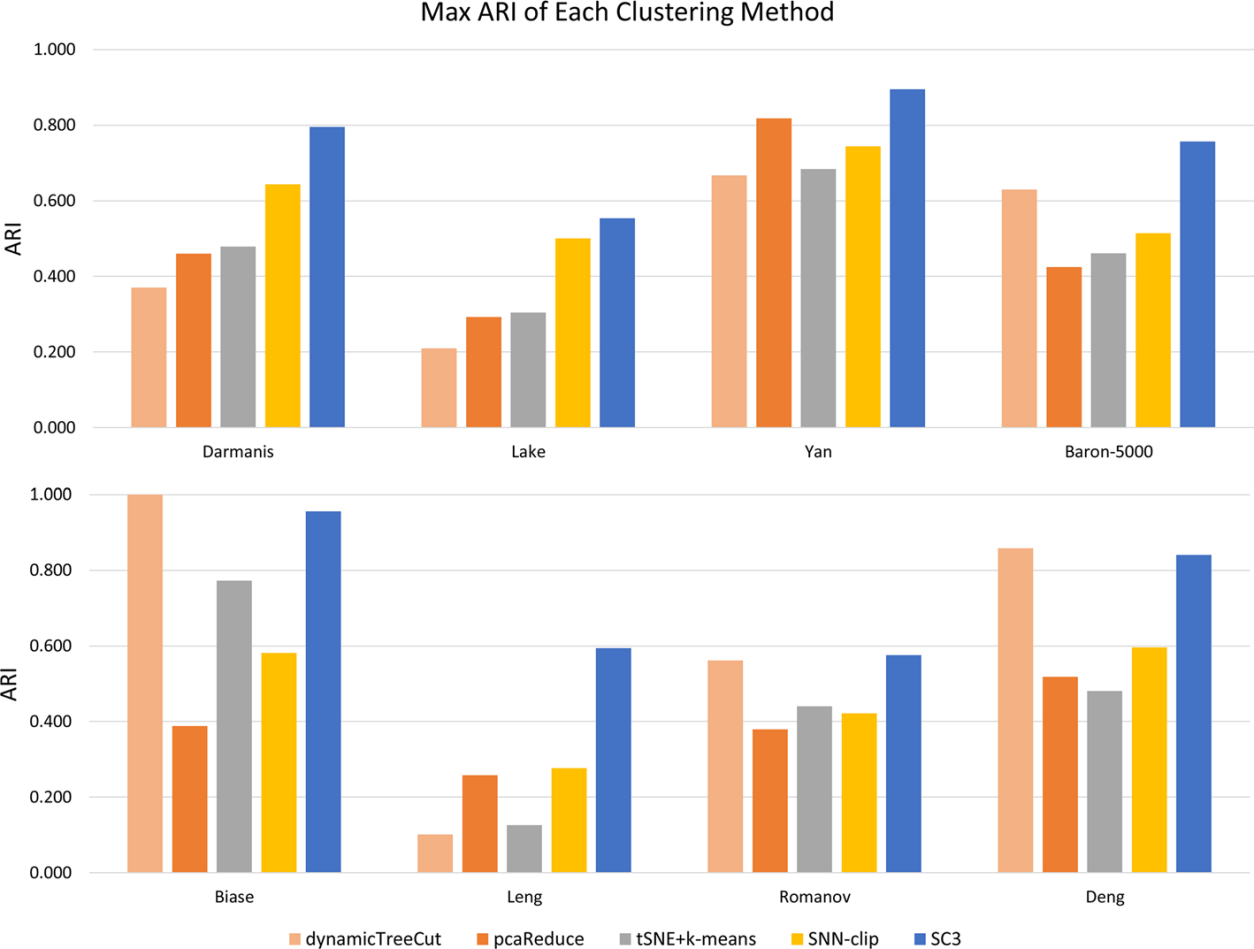
Performance comparisons between SC3-e and the other clustering methods by using the best preprocessing methods

In this section, we compare the performance of SC3-e with the other four clustering methods by using the best preprocessing methods, where SC3-e, pcaReduce and tSNE + *k*-means were run 100 times to capture the average accuracy, while SNN-clip and dynamicTreecut were run only once, as their solutions are stable. After running all the clustering methods on the eight data sets, the results showed that SC3-e performed the best for six data sets, except for the Biase data set and Deng data set, where only the dynamicTreeCut algorithm performed slightly better than SC3-e (see Fig. 3). However, when we ran SC3 by using its default preprocessing method (log transformation), it performed worse than many of the other clustering algorithms by using the best preprocessing method. For example, for the Yan data set, the average accuracies of SC3-e and SC3 were 0.895 and 0.674, respectively, and the accuracies of pcaReduce, SNN-clip and tSNE + kmeans were 0.818, 0.744 and 0.684, all of which were higher than the accuracy of SC3. For the Leng data set, the average accuracies of SC3-e and SC3 were 0.594 and 0.22, respectively, and the accuracies of pcaReduce and SNN-clip were 0.258 and 0.277, respectively, which were also higher than the accuracy of SC3. Therefore, SC3-e performs the best among all the compared clustering methods for almost all the data sets because of its effective and stable discrimination of the best preprocessing method, which significantly enhances the performance of SC3 with its default settings.

## Discussion

The accurate identification of diverse cell types based on noisy scRNA-seq data sets is a highly challenging problem, and many clustering methods have been developed to solve this problem by using different strategies or mathematical models. Most of the clustering methods preprocess the gene/transcript expression data before cell-type clustering, and the most frequently used preprocessing methods are log transformation, z-score transformation, no transformation, and the newly developed method, sctransform. In this study, we found that it is nontrivial to discriminate which preprocessing method is best for a specific clustering algorithm for a given data set because different clustering methods are quite differently impacted by different preprocessing methods for different data sets. log transformation seems to be applicable to the most clustering algorithms, followed by z-score transformation, and the effect of sctransform is very similar to that of log transformation, but the time consumption of sctransform is relatively high. We also found that no specific preprocessing method was applicable to all clustering methods for any given scRNA-seq data set. Moreover, different preprocessing methods will result in quite different clustering results and accuracies. Therefore, it is a highly challenging and important problem to choose an appropriate preprocessing method before cell-type clustering.

Based on such results, we specifically designed the graph-based SC3-e algorithm for discriminating the best data preprocessing method for the SC3 algorithm, which is currently the most frequently used clustering method. This approach first builds two new graph models, a clustering graph and a contracted clustering graph, for each preprocessing method by using the clustering result and the corresponding consensus matrix generated by SC3, based on which the closeness of cells in the same cluster and the closeness of a cluster to the other clusters are effectively measured. Then, a C-score value can be calculated for each preprocessing method based on the two graphs, which is able to discriminate the best preprocessing method effectively and stably for the SC3 clustering algorithm. When tested on eight frequently used single-cell gene expression data sets, SC3-e performed the best among all the other clustering methods for almost all the data sets, which significantly enhanced the performance of SC3 with default settings.

In addition, we evaluated the effects of the three parameters, the number of clusters (*num_clusters*), ${\upalpha }$, and $\upbeta$ on the performance of the algorithm SC3-e. For the parameter *num_clusters*, we tested its effects by setting different values of *num_clusters*. Results showed that changing the parameter *num_clusters* may result in the change of the best preprocessing method. For example, on the Biase data set, the best preprocessing method was z-score transformation if the *num_clusters* was set to 4, while the best preprocessing method was log transformation if the *num_clusters* was set to 3, 5, or 6 (see Additional file 2: Table S2 for more examples). However, under different values of *num_clusters*, the algorithm SC3-e always accurately identified the best preprocessing method (see Additional file 2: Table S2 for details). For the two parameters ${\upalpha }$ and $\upbeta$, reducing the value of ${\upalpha }$ can decrease the connections between cells in the same cluster, and increasing the value of $\upbeta$ can decrease the connections between cells in different clusters. Then we also tested their effects on the performance of the algorithm SC3-e by setting different values. Results showed that the two parameters ${\upalpha }$ and $\upbeta$ only slightly affected the performance of SC3-e, and reducing the value of ${\upalpha }$ or increasing the value of $\upbeta$ tended to obtain a more accurate C-score (see Additional file 3: Table S3 for details). However, too low $\upalpha$ (or too high $\upbeta$) may result in no connections between cells in the same cluster (or no connections between cells in different clusters), and the computed C-score would be 0. Therefore, if the cell number of a specific data set is too small (e.g., no more than 100 cells), a relatively high $\upalpha$ and low $\upbeta$ are recommended in practice.

## Conclusions

To the best of our knowledge, SC3-e is the first algorithm specifically designed for selecting the best preprocessing method before cell-type clustering. And results showed that it can always accurately discriminate the best preprocessing method and therefore largely enhance the clustering performance of the popular algorithm SC3. The software SC3-e has been developed to be user-friendly and is expected to play a crucial role in new discoveries of single-cell clustering using scRNA-seq, especially in complex human diseases such as cancers, the discovery of new cell types, and so on.

## Methods

### Data sets

We collected eight commonly used scRNA-seq data sets in which cell types were known a priori or validated in the respective study for benchmarking the performance of each clustering algorithm under different data preprocessing methods. The eight data sets (see Table 2) came from Darmanis [22], Lake [23], Yan [24], Romanov [25], Baron [26], Biase [27], Deng [28], and Leng [29]. For the Baron data, we randomly selected 5000 cells since SC3 will randomly select 5000 cells from the data if the cell number exceeds 5000, and this data set is referred to as Baron-5000. The numbers of cells, genes, cell types, and normalization units are clearly provided in Table 2.

**Table 2:** Characterization of the eight published scRNA-seq data sets

Data sets	# Cells	# Genes	# Cell types	# Units
Darmanis	466	22,088	9	CPM
Lake	3042	25,051	16	TPM
Yan	90	20,214	6	RPKM
Romanov	2881	24,341	7	READS
Baron-5000	5000	20,125	14	READS
Biase	56	25,734	4	FPKM
Deng	268	22,431	6	RPKM
Leng	460	19,084	4	QN

### Data preprocessing and cell-type clusterings

Five frequently used clustering methods, dynamicTreecut, pcaReduce, tSNE followed by *k*-means clustering (tSNE + *k*-means), SNN-clip, and SC3, and four of the most commonly used preprocessing methods, log transformation, z-score transformation, no transformation, and sctransform, were applied in this study to analyze the performance of different clustering methods under different preprocessing methods. Given input gene expression matrix data in which columns represent cells and rows correspond to genes/transcripts, different transformations were calculated as follows:

$$Input = \left( {\begin{array}{*{20}c} {x_{1,1} } & {x_{1,2} } & \ldots & {x_{1,n} } \\ {x_{2,1} } & {x_{2,2} } & \ldots & {x_{2,n} } \\ \ldots & \ldots & \ldots & \ldots \\ {x_{m,1} } & {x_{m,2} } & \ldots & {x_{m,n} } \\ \end{array} } \right)_{m \times n}$$

$${\text{log transformation}}:x_{i,j} ^{\prime} = \log_{2} (x_{i,j} + 1)$$

$${\text{z-score transformation}}:x_{i,j} ^{\prime} = \frac{{x_{i,j} { - }\mu_{i} }}{{\sigma_{i} }}$$

$$x_{i,j} ^{\prime} = \frac{{x_{i,j} { - }\mu_{i} }}{{\sigma_{i} }}$$

where ${\mu }_{i}$ is the average of the *i-*th row of the input data matrix, and ${\sigma }_{i}$ is the standard deviation of the *i-*th row of the input data matrix.

After transformations, four preprocessed matrices were generated, referred to as log data (log transformation), z-score data (z-score transformation), no data (no transformation), and sctransform data (sctransform). Then, the applied clustering methods took each of these four data matrices as the input to perform cell-type clustering. To obtain stable clustering solutions for the clustering methods, we ran pcaReduce, tSNE + *k*-means and SC3 50 times, while SNN-clip and dynamicTreecut were run only once since their solutions are stable. Then, the clustering accuracy was calculated according to the commonly used criterion of the adjusted Rand index (ARI) [30] as follows.

$$ARI = \frac{{\sum\nolimits_{ij} {\left( {\begin{array}{*{20}c} {n_{ij} } \\ 2 \\ \end{array} } \right) - \left[ {\sum\nolimits_{i} {\left( {\begin{array}{*{20}c} {a_{i} } \\ 2 \\ \end{array} } \right)\sum\nolimits_{j} {\left( {\begin{array}{*{20}c} {b_{j} } \\ 2 \\ \end{array} } \right)} } } \right]/\left( {\begin{array}{*{20}c} n \\ 2 \\ \end{array} } \right)} }}{{\frac{1}{2}\left[ {\sum\nolimits_{i} {\left( {\begin{array}{*{20}c} {a_{i} } \\ 2 \\ \end{array} } \right) + \sum\nolimits_{j} {\left( {\begin{array}{*{20}c} {b_{j} } \\ 2 \\ \end{array} } \right)} } } \right] - \left[ {\sum\nolimits_{i} {\left( {\begin{array}{*{20}c} {a_{i} } \\ 2 \\ \end{array} } \right)\sum\nolimits_{j} {\left( {\begin{array}{*{20}c} {b_{j} } \\ 2 \\ \end{array} } \right)} } } \right]/\left( {\begin{array}{*{20}c} n \\ 2 \\ \end{array} } \right)}}$$

where *n*_*ij*_ represents values from the contingency table, *a*_*i*_ is the sum of the *i*th row of the contingency table, and *b*_*j*_ is the sum of the *j*th column of the contingency table.

### The SC3-e algorithm

**Fig. 4:**
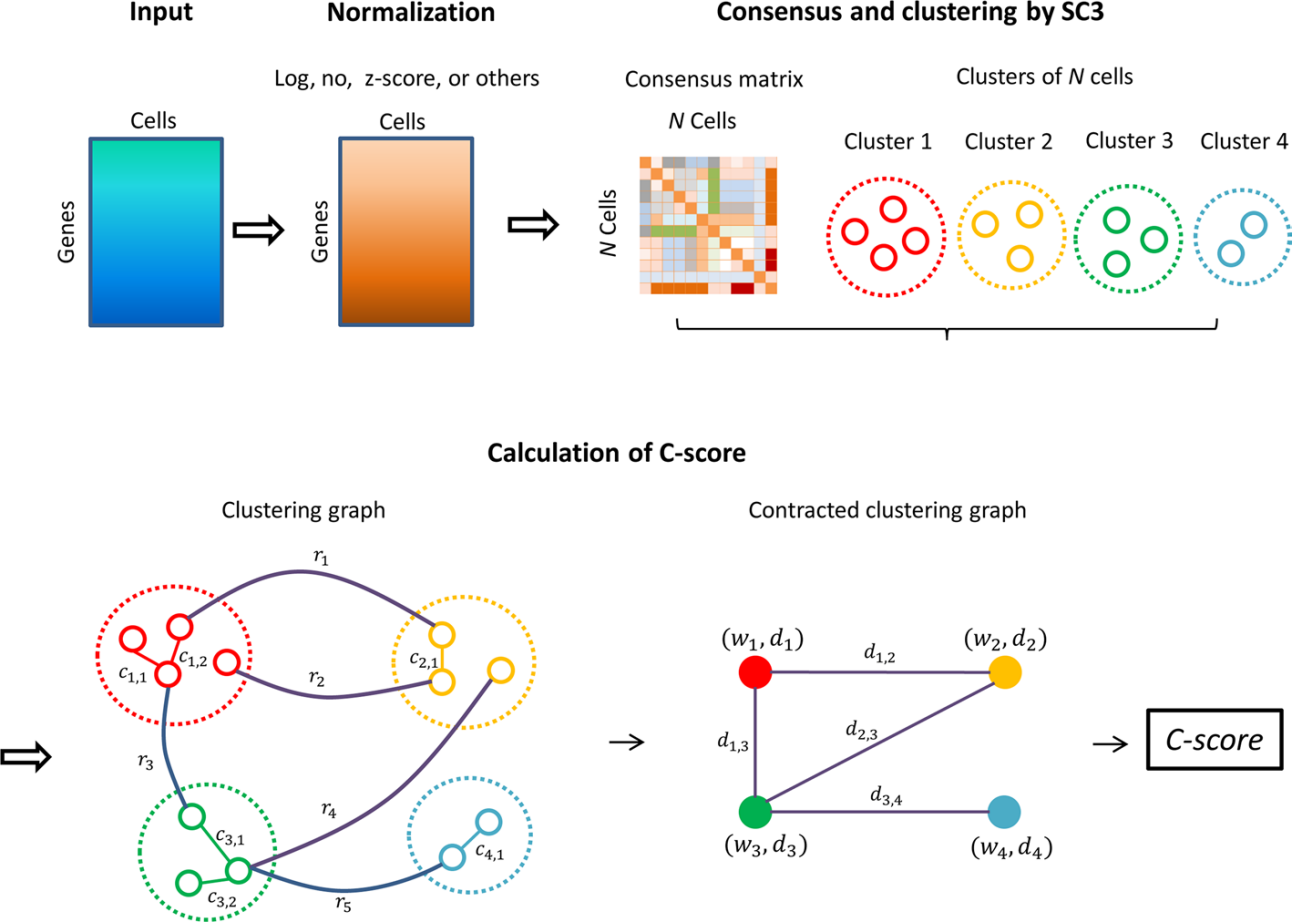
Flowchart of SC3-e

To select the best preprocessing method for each given data set for the SC3 clustering algorithm, we designed the graph-based SC3-e algorithm (see Fig. 4 for the pipeline of SC3-e), which significantly enhances the performance of SC3.

#### Running SC3 under different preprocessing methods

Given a gene expression data set, *M*, in which rows represent genes and columns correspond to cells, four kinds of transformations (log transformation, z-score transformation, no transformation, and sctransform) were performed on *M*, and four preprocessed matrices, *M*-log, *M*-zscore, *M*-no, and *M*-sctransform were generated. Then, the SC3 pipeline was applied to each of the preprocessed matrices to produce a consensus matrix, *C*, and a clustering result, *T*. The value of *c*_*ij*_ (*c*_*ij*_ belongs to the interval [0, 1]) in the consensus matrix, *C*, represents the similarity between cells *i* and *j*, and the larger the value, the more similar they are. Therefore, we finally obtained four consensus matrices, *C*-log, *C*-zscore, *C*-no, and *C*-sctransform, and four corresponding clustering results, *T*-log, *T*-zscore, *T*-no, and *T*-sctransform.

#### Building the clustering graphs

To discriminate the best clustering result from *T*-log, *T*-zscore, *T*-no, and *T*-sctransform, based on the consensus matrices *C*-log, *C*-zscore, *C*-no, and *C*-sctransform, we first built a clustering graph for each of the four clustering results. Given a clustering result, *T* and its corresponding consensus matrix, *C*, each node in the clustering graph represents a cell in the data set, and an in-cluster edge is added between two nodes, *n*_*i*_ and *n*_*j*_, if and only if the two corresponding cells, *i* and *j*, are in the same cluster and their consensus value, *c*_*ij*_, is lower than a threshold of ${\upalpha }$ (default value 0.6), and the edge is labeled as *c*_*m,n*_, where *m* indicates that this edge belongs to the *m-*th cluster, and *n* means that it is the *n-*th in-cluster edge in the cluster. At the same time, an out-cluster edge is added between two nodes, *n*_*i*_ and *n*_*j*_, if and only if the two corresponding cells, *i* and *j*, are in two different clusters and their consensus value, *c*_*ij*_, is higher than a threshold of $\upbeta$ (default value 0.5), and the edge is labeled *r*_*p*_, where *p* represents the *p-*th out-cluster edge in the clustering graph. After the processing of each clustering result, we finally obtained four clustering graphs *G*-log, *G*-zscore, *G*-no, and *G*-sctransform.

#### Contraction of the clustering graphs

After constructing a clustering graph, *G*, for each clustering result, we contracted the nodes in the same cluster into a single node, referred to as a cluster node, and two cluster nodes, *v*_*i*_ and *v*_*j*_, are connected by an edge if and only if there is at least one out-cluster edge between the two corresponding clusters in the original clustering graph. Each edge in the contracted clustering graph is weighted by the value of *d*_*i,j*_, calculated as follows:

$$d_{i,j} = \frac{{\sum\nolimits_{{r_{p} \in E_{i,j} }} {(r_{p} - \beta )} }}{{\left| {E_{i,j} } \right|}}$$

where *E*_*i,j*_ represents the set of out-cluster edges between clusters *i* and *j* in the original clustering graph, and |*E*_*i,j*_| represents the number of edges in set *E*_*i,j*_. According to the definition, a smaller *d*_*i,j*_ clearly indicates lower closeness between two clusters, *i* and *j*. Then, each node in the contracted clustering graph is weighted by two values, *w*_*i*_ and *d*_*i*_, where *w*_*i*_ measures the closeness among cells in the same cluster, *i*; *d*_*i*_ measures the closeness between cluster *i* and all the other clusters; and the two values *w*_*i*_ and *d*_*i*_ are defined as follows:

$$w_{i} = \left\{ {\begin{array}{*{20}l} {\frac{{\sum\nolimits_{j = 1, \ldots ,k} {(\alpha - c_{i,j} )} }}{k}} ,& {k \ne 0}\\ 0, & {k = 0} \end{array}} \right.$$

where *k* is the number of edges in cluster *i*.

$$d_{i} = \left\{ {\begin{array}{*{20}l} {\sum\nolimits_{{j \in D(v_{i} )}} {d_{i,j} } }, & {\left| {D(v_{i} )} \right| \ne 0} \\ 0, & {\left| {D(v_{i} )} \right| = 0} \end{array} } \right.$$

where *D*(*v*_*i*_) represents the set of the neighbors of cluster node *v*_*i*_.

#### Selection of the best preprocessing method for SC3

To choose the best data preprocessing method for SC3 based on the contracted clustering graphs, we calculate a C-score value as follows:

$$W_{in} = \frac{{\sum\nolimits_{i = 1, \ldots ,n} {w_{i} } }}{n}$$

$$D_{out} = \frac{{\sum\nolimits_{i = 1, \ldots ,n} {d_{i} } }}{n}$$

$$C\text{-}score = W_{in} \cdot D_{out}$$

where *n* represents the number of clusters, and *w*_*i*_ and *d*_*i*_ represent the two corresponding weights of cluster node *v*_*i*_. According to the above definition, a lower value of the C-score means that cells in the same cluster show closer relationships, while cells in different clusters show more distant relationships. Therefore, the smallest value of the C-score demonstrates the best data preprocessing for SC3 and therefore the best cell-type clustering.

SC3 is an unstable algorithm that may generate different clustering results and consensus matrices when it is run multiple times and therefore produces different C-scores. To obtain a stable C-score under each transformation, we can repeat the whole process *N* times (the default value of *N* is 1) and generate *N* C-scores for each transformation; then, the final stable C-score can be obtained by computing the average of the *N* C-scores.

## Supplementary information

Additional file 1: Table S1. This file contains 50 pairs of ARI and C-score values generated by running SC3 50 times on each data set.

Additional file 2: Table S2. This file contains the ARI and C-score values under different values of the parameter num_cluster on each data set.

Additional file 3: Table S3. This file contains the ARI and C-score values under different values of the two parameters α, and β on each data set.

## Data Availability

The source code of SC3-e and the eight data sets in this study are available at https://sourceforge.net/projects/transcriptomeassembly/files/.
